# Peripheral and Autonomic Neuropathy Status of Young Patients With Type 1 Diabetes Mellitus at the Time of Transition From Pediatric Care to Adult-Oriented Diabetes Care

**DOI:** 10.3389/fendo.2021.719953

**Published:** 2021-08-27

**Authors:** Anna Vágvölgyi, Ágnes Maróti, Mónika Szűcs, Csongor Póczik, Dóra Urbán-Pap, István Baczkó, Attila Nemes, Éva Csajbók, Krisztián Sepp, Péter Kempler, Andrea Orosz, Tamás Várkonyi, Csaba Lengyel

**Affiliations:** ^1^Department of Medicine, University of Szeged, Szeged, Hungary; ^2^Department of Pediatrics and Pediatric Health Center, University of Szeged, Szeged, Hungary; ^3^Department of Medical Physics and Informatics, University of Szeged, Szeged, Hungary; ^4^Department of Pharmacology and Pharmacotherapy, University of Szeged, Szeged, Hungary; ^5^Department of Pharmacology and Pharmacotherapy, Interdisciplinary Excellence Centre, University of Szeged, Szeged, Hungary; ^6^Department of Oncology and Internal Medicine, Semmelweis University, Budapest, Hungary

**Keywords:** health care transition, autonomic neuropathy, type 1 diabetes mellitus, peripheral sensory neuropathy, blood pressure

## Abstract

**Introduction:**

The prevalence of neuropathic lesions in young patients with type 1 diabetes mellitus (T1DM) at the time of transition from pediatric care to adult-oriented diabetes care is poorly studied. A comparative study with healthy volunteers to assess the possible neuropathic condition of this special population and to identify the potential early screening needs has not been performed yet. The results may provide important feedback to pediatric diabetes care and a remarkable baseline reference point for further follow up in adult diabetes care.

**Patients and Methods:**

Twenty-nine young patients with T1DM [age: 22.4 ± 2.9 years; HbA1c: 8.5 ± 2.1%, diabetes duration: 12.2 ± 5.8 years; (mean ± SD)] and 30 healthy volunteers (age: 21.5 ± 1.6 years; HbA1c: 5.3 ± 0.3%) were involved in the study. Autonomic function was assessed by standard cardiovascular reflex tests. Complex peripheral neuropathic testing was performed by Neurometer^®^, Neuropad^®^-test, Tiptherm^®^, Monofilament^®^, and Rydel-Seiffer tuning fork tests.

**Results:**

T1DM patients had significantly higher diastolic blood pressure than controls (80 ± 9 *vs.* 74 ± 8 mmHg, p < 0.01), but there was no significant difference in systolic blood pressure (127 ± 26 *vs.* 121 ± 13 mmHg). Cardiovascular reflex tests had not revealed any significant differences between the T1DM patients and controls. No significant differences with Neurometer^®^, Neuropad^®^-test, and Monofilament^®^ were detected between the two groups. The vibrational sensing on the radius on both sides was significantly impaired in the T1DM group compared to the controls with Rydel-Seiffer tuning fork test (right: 7.5 ± 1.0 *vs.* 7.9 ± 0.3; left: 7.5 ± 0.9 *vs.* 7.9 ± 0.3, p < 0.05). The Tiptherm^®^-test also identified a significant impairment in T1DM patients (11 sensing failures *vs.* 1, p < 0.001). In addition, the neuropathic complaints were significantly more frequently present in the T1DM patient group than in the controls (9 *vs.* 0, p < 0.01).

**Conclusion:**

In this young T1DM population, cardiovascular autonomic neuropathy and cardiac morphological alterations could not be found. However, Rydel-Seiffer tuning fork and Tiptherm^®^-tests revealed peripheral sensory neurological impairments in young T1DM patients at the time of their transition to adult diabetes care.

## Introduction

Diabetes mellitus is one of the most common chronic diseases in children and adolescents (1, 2), and more than 90% of all diabetes cases in childhood is type 1 diabetes mellitus (T1DM) (3). According to a nationwide, population-based Hungarian database of children and adolescents constructed between 2001 and 2016, the incidence of T1DM in Hungary increased from 16/100,000 to 23/100,000 and the prevalence of T1DM from 114/100,000 to 209/100,000 with a male predominance (4). Several complications, including retinopathy, diabetic kidney disease, hypertension, cardiovascular autonomic, and peripheral sensory neuropathy, are associated with the onset of diabetes in childhood and adolescence and represent a significant burden for the health care system (5, 6). Diabetic autonomic neuropathy was defined as an autonomic nervous system disorder in diabetes or prediabetes after the exclusion of other causes, while diabetic peripheral neuropathy as a “symmetrical, length-dependent sensorimotor polyneuropathy attributable to metabolic and microvessel alterations as a result of chronic hyperglycemia exposure (diabetes) and cardiovascular risk covariates” (7).

The prevalence of cardiovascular autonomic neuropathy (CAN) was at least 20% in unselected type 1 and type 2 diabetic patients (8–10) and very low in newly diagnosed patients with T1DM (11–13). However, the prevalence of CAN increases substantially with age and diabetes duration both in T1DM—as at least 30% prevalence was observed in the Diabetes Control and Complications Trial (DCCT) and the Epidemiology of Diabetes Interventions and Complications (EDIC) follow-up study after 20 years of diabetes duration (14)—and in type 2 diabetes mellitus (T2DM) it may be up to 60% after 15 years (8, 13, 15, 16). CAN is a proven risk factor for cardiovascular morbidity, and its presence causes a 3.65-fold increase in the relative risk of mortality (8, 10, 12).

Clinical diabetic neuropathy is rarely seen in pediatric populations; however, subclinical neuropathy (peripheral nerve function abnormalities without any clinical symptoms) may be common among adolescents (17–20). In diabetic peripheral neuropathy (DPN), the dysfunction of the small and/or large nerve fibers can be seen, and DPN is associated with neuropathic pain, foot ulceration, gangrene (21). Several studies showed that the prevalence of DPN was 7–11% in young patients (8–21 years old) with T1DM (22, 23). Risk factors for DPN in youth with T1DM are poor glycemic control, large glycemic variability, older age, pubertal stage, longer diabetes duration, smoking, increased diastolic blood pressure, obesity, increased LDL cholesterol and triglyceride, and lower HDL cholesterol levels (23–30). Subclinical DPN has been also found in children with good metabolic control and short diabetes duration, which suggests an additional role of genetic predisposition (22, 26, 31).

Health care transition (HCT) is a prepared, systematic process to transfer adolescents and young adults with chronic diseases from pediatric to adult health care systems (32, 33); nevertheless, young T1DM patients have to face unique challenges regarding lifelong diabetes management skills (34). A recent review by Schmidt et al. (35) showed that the structured, professionally guided HCT process (planning, transfer assistance, and integration) resulted in improvements in disease-specific measures, quality of life, and self-care skills. Several studies demonstrated an improvement in the glycemic control of young T1DM patients following a structured transition program from pediatric to adult diabetes care (36–39); however, the prevalence of neuropathic lesions in young patients with T1DM at the time of transition is not known. Our goal was to assess the possible neuropathic condition of this special population at the time of transition to define the potential earlier screening needs. The present results may provide important feedback to pediatric diabetes care and a remarkable baseline reference point for further follow-up in adult diabetes care.

## Materials and Methods

### Study Population

Young patients with type 1 diabetes mellitus were eligible for the study at the time of health care transition from the Department of Pediatrics and Pediatric Health Center to the Department of Medicine at the University of Szeged, Hungary. The measurements were performed from September 2019 to February 2020 as the first neuropathic status assessment in adult care. The exclusion criteria included cases of pernicious anemia, alcoholism, chronic hepatitis, uremia, exposure to chemical agents, nerve compression, and chemotherapy treatment.

We studied 29 young patients with T1DM [age: 22.4 ± 2.9 years; body mass index (BMI): 22.8 ± 3.0 kg/m^2^; hemoglobin A1c (HbA1c): 8.5 ± 2.1%, mean diabetes duration: 12.2 ± 5.8 years; 13 men/16 women; (mean ± SD)]. A total of 30 age-matched healthy volunteers (age: 21.5 ± 1.6 years; BMI: 22.3 ± 3.7 kg/m^2^; HbA1c: 5.3 ± 0.3%; 12 men/18 women) were enrolled in the study as controls.

### Cardiovascular Autonomic Function Testing

Standard cardiovascular reflex tests were applied (40) to characterize the presence and severity of the cardiovascular autonomic neuropathy. These measurements provide a non-invasive, clinically relevant, reproducible, and standardized gold-standard determination of autonomic function (13). During the cardiovascular reflex tests, blood pressure was measured and six-lead electrocardiograms (ECG) were continuously recorded. The ECG signals were digitized at 2 kHz sampling rate with a multichannel data acquisition system (Cardiosys-A01 software, MDE Heidelberg GMBH, Heidelberg, Germany) connected to a personal computer and stored for later offline analysis.

Three of these reflex tests record the changes of heart rate during specific maneuvers: during deep breathing (HRRDB), in positions of lying then standing up (30/15 ratio), and during and after a Valsalva maneuver (Valsalva ratio [VR]), while one test was designed to evaluate systolic blood pressure changes from lying to standing up (SBPRSU) (8). Those tests aiming to detect changes in heart rate are used primarily (but not exclusively) for the assessment of parasympathetic innervation, while the blood pressure response predominantly reflects the impairment of sympathetic function (41).

#### Heart Rate Response to Deep Breathing

Normally, the heart rate is increased during inspiration and decreased by expiration. The patient was asked to breathe deeply at the rate of six breaths per minute (5 s in and 5 s out). The result was expressed as the difference between the measured maximum and minimum heart rates (beats/min) during the six breathing cycles.

#### Heart Rate Response to Standing Up (30/15 Ratio)

Following the position change from lying to standing up, the heart rate is immediately increased and reaches its maximum at around the 15^th^ beat after standing up. Then, a relative bradycardia occurs in healthy subjects with the lowest heart rate at about the 30^th^ heartbeat. At the start of the test, the patient was in the supine position while the electrocardiogram was recorded continuously. Then the patient stood up without interrupting the recording. The 30/15 ratio was expressed as the ratio of the longest R-R interval (at around the 30^th^ beat) to the shortest R-R interval (at around the 15^th^ beat) following standing up.

#### Heart Rate Response to Valsalva Maneuver (Valsalva Ratio)

During the strain period of Valsalva maneuver, the blood pressure drops and the heart rate rises under physiologic conditions. Following the procedure, the blood pressure rises and the heart rate slows. The patient was instructed to blow into a mouthpiece connected to a modified manometer and holding it at a pressure of 40 mmHg for 15 s, while an electrocardiogram was recorded continuously. The Valsalva ratio was calculated as the ratio of the longest R-R interval after the maneuver to the shortest R-R interval during the procedure.

#### Systolic Blood Pressure Response From Lying to Standing Up

In healthy standing subjects, the pooling of blood in the lower extremities is rapidly corrected by peripheral vasoconstriction. Severe postural hypotension is a characteristic sign of CAN. This test is based on blood pressure measurements in lying position and following standing up. The postural fall in blood pressure is defined as the difference between systolic blood pressure after 10 min in the supine position and systolic blood pressures at the 1^st^, 5^th^, and 10^th^ minutes after standing up. The largest difference from the systolic pressure in lying is evaluated as the blood pressure response to standing.

### Sensory Nerve Testing

The peripheral sensory function was studied with a Neurometer^®^ (NM-01/CPT Neurometer, MDE Heidelberg GmbH, Heidelberg, Germany). This device is suitable for the quantification of the function of different nerve fibers and provides a simple, non-invasive, and quantitative measure of peripheral sensory function (42). Low voltage, electric sine-wave stimulation was applied transcutaneously, and the current perception threshold (CPT) values were determined. In our study, the median and peroneal nerves were tested. The surface electrodes with 1 cm diameter were placed on the terminal phalanx of the index finger and the great toe. The electrodes were fixed only on intact skin surface, while wounds or scars would have disturbed the peripheral sensations. The amplitude of the delivered stimuli was between 0.01 and 9.99 mA. The stimulus was initially increased until a sensation was reported, then short stimuli (2–5 s) were applied at progressively lower amplitudes until a minimal threshold for consistent detection was determined. The CPT values of the upper and lower limbs were detected at three different stimulating frequencies (2000, 250, 5 Hz). Thus, the thick myelin sheath coated (2 kHz), thin myelinated (250 Hz), and thin non-myelinated fibers (5 Hz) were selectively tested.

Neuropad^®^ screening tests (43) were applied by each candidate to detect the sudomotoric dysfunction, a component of autonomic neuropathy. The Neuropad^®^ screening test has a very high sensitivity for the detection of diabetic peripheral neuropathy (44) and works on the basis that damage to the nerve fibers in the feet leads not only to a loss of sensation but also to a malfunctioning sweat system and, in turn, to unusually dry skin on the feet. The adhesive pad contains the blue salt anhydrous cobalt II chloride, which reacts and changes to pink when exposed to water. Patients were allowed a 10-min relaxing period in definite room temperature (23°C) after they had removed their shoes and socks. Tests were applied to both soles at the level of the first through second metatarsal heads. The time to color change was exactly 10 min after application. Full pink end-color was normal, mixed pink with blue as pending, total blue was evaluated as pathologic.

The 128 Hz Rydel-Seiffer graduated tuning fork test was applied to evaluate the vibration perception on the distal end of both radius and hallux on both sides. Interpretation of the tuning fork results was based on age-dependent normal values previously published by Martina et al. in 1998 (45). By reading the result on the scale of 8, 7-8 were defined as normal, 6 as borderline and 1-5 were evaluated as pathologic which refers to vibration sensory impairment.

The Semmes-Weinstein Monofilament Test^®^ with a 10 g monofilament is an objective and simple instrument used in screening the diabetic foot for loss of protective sensation (46). A properly calibrated device was used in a quiet and relaxed setting, and the candidates were not able to see if and where the examiner applies the filament. Five areas were tested in all cases on the sole: the hallux, first metatarsus, second metatarsus, and third and fifth metatarsus heads. Normal sensation in four to five areas was defined as normal, and normal sensation just in zero to three areas was evaluated as pathologic.

The Tiptherm^®^ (Tip-Therm GmbH, Düsseldorf, FRG) is a pen-shaped device with two flat sides used for early diagnosis of symmetrical polyneuropathy, which measures temperature sensitivity of the skin (47). This device consists of a plastic cylinder on one end and a metal cylinder on the other end, with a diameter of 14 mm each. Each end is applied in a random order to the skin for 1 s at both hands and feet. The tested person has to decide which of the two touches feels cooler (48). Candidates with normal temperature sensation (<10°C) recognize the different subjective temperature sensation of the two flat surfaces with Tiptherm^®^. Candidates with disturbed temperature sensation (≥10°C) are unable to recognize the difference.

Neuropathic complaints were assessed by a questionnaire. Every individual had to make a statement about the presence or absence of burning, pinprick sensation, numbness, tingling, hypoesthesia, hyperesthesia, and also about the intensity of these symptoms and frequency of their occurrence.

### Laboratory Data Collection

Fasting venous blood and urine samples were obtained from patients and controls for the determination of glucose, hemoglobin A1c, blood urea nitrogen, corrected calcium, magnesium, sodium, potassium, total protein, albumin, alkaline phosphatase, urine pH, and for complete lipid, liver, and blood test profiles. Due to logistical reasons, the collection of the blood and urine samples was not possible from all patients and controls.

### Echocardiographic Examination

Transthoracic echocardiography has been performed to determine standard morphological and functional parameters. The subjects underwent a complete two-dimensional (2D) transthoracic echocardiographic study using a Toshiba Artida imaging system (Toshiba Medical Systems, Tokyo, Japan) with a PST-30SBP phased-array transducer (1–5 MHz). The images were obtained in accordance with the recommendations of the American Society of Echocardiography and the European Association of Cardiovascular Imaging (49). In all cases, left ventricular (LV) dimensions, volumes and ejection fraction (EF), and left atrial (LA) dimensions were measured, and complete 2D Doppler studies were performed. For purely logistical reasons, not all T1DM patients and controls were subjected to echocardiography.

### Statistical Analysis

Statistical data were reported as the mean ± SD, with frequencies (n) and percentages (%), when appropriate. Power analysis for the study was performed using the software G* Power (Version 3.1.9.2) for power-and-sample size calculation (University of Düsseldorf, Germany). The calculated sample size was 28, working with an effect size d = 0.8, alpha as Type I error of 0.05, and a power value of 0.95. Pearson’s chi-squared test or Fisher’s exact test was used to analyze categorical data, whereas independent samples t-test was used in case of continuous data. Statistical tests were performed using R statistical software (R version 3.6.2, https://www.r-project.org/), and values of p < 0.05 were considered significant.

### Ethics Statement

This study was carried out in accordance with the Declaration of Helsinki (2000) of the World Medical Association and was approved by the Hungarian Medical Research Council (approval No. 31891-5/2019/EÜIG). All subjects have given written informed consent of the study.

## Results

### Clinical and Laboratory Data of Young Patients With Type 1 Diabetes Mellitus and Control Subjects

**Figure 1:**
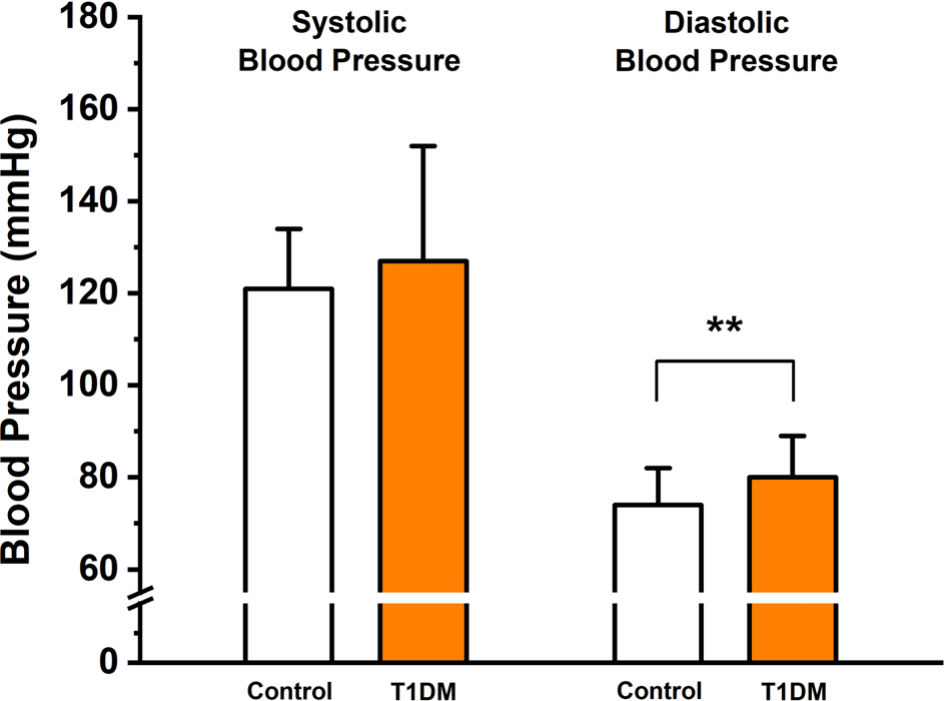
Mean systolic and diastolic blood pressure in the control group and in type 1 diabetes mellitus (T1DM) patients; **p<0.01 *vs.* control group; n=30 and 29 individuals in the control and T1DM groups, respectively.

Relevant clinical data of T1DM patients and control subjects are presented in **Table 1**. The mean duration of type 1 diabetes was 12.2 ± 5.8 years. Age, weight, height, body mass index, and waist-to-hip ratio did not differ significantly between control subjects and young diabetic patients. Mean systolic blood pressure did not differ significantly between T1DM patients and control subjects; however, T1DM patients had higher diastolic blood pressure (80 ± 9 *vs.* 74 ± 8 mmHg; p = 0.003; **Figure 1**). At the time of transition, 10 young T1DM patients received multiple injections and 19 T1DM patients were on insulin pump therapy. Only two of the T1DM individuals used continuous glucose monitoring system (CGMS) both as augmentation for their insulin pump therapy. After the transition of the T1DM patients, in the adult-oriented diabetes care in the terms of multiple injections or pump therapy, none of the patients’ therapy has changed over the past year; however, since then nine more patients started to use CGMS. Among 29 T1DM patients, two patients had hypercholesterolemia and six patients were receiving antihypertensive medications (two were taking beta blockers, five patients were taking angiotensin-converting enzyme inhibitors, or angiotensin receptor blockers, and three T1DM patients were taking Ca^2+^ channel blockers).

**Table 1:** Clinical data in the study groups.

	T1DM patients (n = 29)	Controls (n = 30)	p value
**Diabetes duration (years)**	12.2 ± 5.8		
**Age (years)**	22.4 ± 2.9	21.5 ± 1.6	0.115
**Weight (kg)**	66 ± 13	68 ± 14	0.622
**Height (cm)**	170 ± 11	174 ± 9	0.113
**BMI (kg/m^2^)**	22.8 ± 3.0	22.3 ± 3.7	0.58
**Waist-to-hip ratio**	0.79 ± 0.07	0.80 ± 0.14	0.891
**Male sex (%)**	13 (44.8)	12 (40.0)	0.795
**Systolic BP (mmHg)**	127 ± 25	121 ± 13	0.253
**Diastolic BP (mmHg)**	80 ± 9	74 ± 8	***0.003***
**Hypertension (%)**	6 (20.7)	0 (0.0)	***0.011***
**Hypercholesterolemia (%)**	2 (6.9)	0 (0.0)	0.237
**Smoking history (%)**	9 (31)	4 (13.3)	0.125
**Alcohol consumption (%)**	8 (27.6)	13 (43.3)	0.279

The data are presented as mean ± SD. BMI, body mass index; BP, blood pressure. The p values in bold are considered significant.

Regarding the laboratory parameters, average serum glucose and HbA1c values were significantly higher in young patients with type 1 DM compared to healthy controls (**Table 2**). The serum magnesium, albumin, and creatinine levels were significantly lower among T1DM patients, and significantly higher serum alkaline phosphatase level and eGFR were detected in the T1DM patients compared to controls (**Table 2**). Other laboratory parameters (lipid, liver, and blood test profile) did not show any significant differences. Two-dimensional echocardiography has not revealed any significant differences between the two groups. Relevant results are shown in **Table 3**.

**Table 2:** Laboratory data in the study groups.

	T1DM patients	Controls	p value
**Glucose (mmol/L)**	8.4 ± 5.6 (n=18)	4.6 ± 0.6 (n=21)	***0.010***
**HbA1c (%)**	8.5 ± 2.1 (n=23)	5.3 ± 0.3 (n=21)	***<0.001***
**Blood urea nitrogen (mmol/L)**	4.4 ± 1.2 (n=21)	4.3 ± 1.0 (n=22)	0.839
**Corrected calcium (mmol/L)**	2.3 ± 0.1 (n=13)	2.3 ± 0.1 (n=20)	0.373
**Magnesium (mmol/L)**	0.8 ± 0.1 (n=11)	0.9 ± 0.1 (n=19)	***0.031***
**Sodium (mmol/L)**	139.5 ± 2.2 (n=22)	140.1 ± 3.0 (n=21)	0.431
**Potassium (mmol/L)**	4.2 ± 0.3 (n=22)	4.3 ± 0.4 (n=21)	0.776
**Blood urea nitrogen (mmol/L)**	4.4 ± 1.2 (n=21)	4.3 ± 1.0 (n=22)	0.839
**Creatinine (μmol/L)**	70.7 ± 11.8 (n=21)	79.9 ± 16.6 (n=21)	***0.047***
**eGFR (ml/min/1.73 m^2^)**	113.9 ± 22.0 (n=21)	100.8 ± 17.8 (n=21)	***0.040***
**Cholesterol (mmol/L)**	4.7 ± 1.4 (n=22)	4.3 ± 0.9 (n=21)	0.284
**Triglyceride (mmol/L)**	1.3 ± 1.1 (n=22)	1.0 ± 0.5 (n=21)	0.323
**HDL-cholesterol (mmol/L)**	1.6 ± 0.3 (n=21)	1.7 ± 0.4 (n=21)	0.491
**LDL-cholesterol (mmol/L)**	2.2 ± 0.6 (n=17)	2.2 ± 0.7 (n=21)	0.876
**Total protein (g/L)**	74.1 ± 4.4 (n=14)	73.9 ± 8.5 (n=21)	0.93
**Albumin (g/L)**	47.7 ± 5.1 (n=19)	51.4 ± 3.3 (n=21)	***0.012***
**Alkaline phosphatase (U/L)**	97.8 ± 44.5 (n=21)	63.8 ± 13.8 (n=22)	***0.003***
**Urine pH**	6.11 ± 0.8 (n=13)	6.3 ± 0.8 (n=21)	0.493

The data are presented as mean ± SD. HbA1c, hemoglobin A1c. The p values in bold are considered significant.

**Table 3:** Echocardiographic parameters of the study groups.

	T1DM patients (n = 24)	Controls (n = 15)	p value
**Ao (mm)**	27.2 ± 3.0	27.2 ± 2.6	0.978
**LA (mm)**	36.1 ± 4.4	36.7 ± 4.3	0.672
**LVEDD (mm)**	44.9 ± 4.8	46.7 ± 4.0	0.217
**LVESD (mm)**	27.9 ± 2.7	28.9 ± 3.5	0.347
**IVSd (mm)**	8.2 ± 1.0	8.7 ± 0.9	0.099
**PW (mm)**	8.3 ± 1.1	8.6 ± 0.8	0.261
**LVEDV (mL)**	100.0 ± 15.5	101.7 ± 23.2	0.800
**LVESV (mL)**	31.3 ± 6.1	33.1 ± 12.0	0.592
**EF (%)**	69.4 ± 3.3	68.5 ± 4.3	0.491

The data are presented as mean ± SD. Ao, aortic diameter; LA, left atrial diameter; LVEDD, left ventricular end-diastolic diameter; LVESD, left ventricular end-systolic diameter; IVSd, interventricular septum thickness at end-diastole; PW, left ventricular posterior wall thickness at end-diastole; LVEDV, left ventricular end-diastolic volume; LVESV, left ventricular end-systolic volume; EF, ejection fraction.

### Cardiovascular Autonomic Function

Standard cardiovascular reflex tests did not indicate any significant deteriorations in the heart rate responses to deep breathing (HRRDB), to standing up (30/15 ratio), and to Valsalva maneuver (Valsalva ratio, VR) or in the systolic blood pressure response from lying to standing up (SBPRSU) in young T1DM subjects compared to controls. Autonomic parameters of young T1DM patients and age-matched control subjects are shown in **Table 4**.

**Table 4:** Results of the cardiovascular autonomic function tests in T1DM patients and controls.

	T1DM patients (n = 29)	Controls (n = 30)	p value
**HRRDB (1/min)**	32 ± 11	32 ± 9	0.877
**30/15 ratio**	1.2 ± 0.3	1.1 ± 0.2	0.171
**VR**	2.2 ± 0.4	2.3 ± 0.3	0.149
**SBPRSU (mmHg)**	5 ± 6	3 ± 4	0.272

The data are presented as mean ± SD. HRRDB, the heart rate response to deep breathing; 30/15 ratio, the heart rate responses to standing up; VR (Valsalva ratio), the heart rate responses to Valsalva maneuver; SBPRSU, the systolic blood pressure response from lying to standing up.

### Peripheral Sensory Function in Young T1DM Patients and Control Subjects

**Figure 2:**
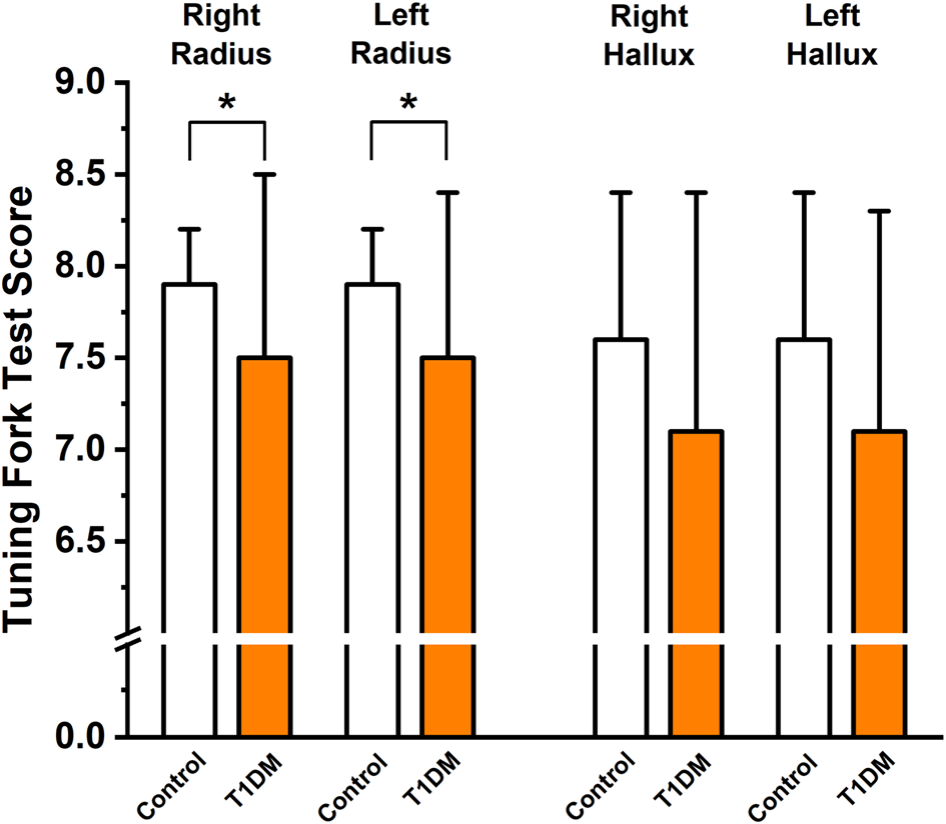
Peripheral sensory function testing using the 128 Hz Rydel-Seiffer graduated tuning fork on the distal end of right (RR), left (LR) radius, and the right (RH) and left (LH) hallux in the control group and in type 1 diabetes mellitus (T1DM) patients; *p<0.05 *vs.* control group; n=30 and 29 individuals in the control and T1DM groups, respectively.

No significant differences were detected with Neurometer^®^ (**Table 5**), Neuropad^®^-test, and Semmes-Weinstein Monofilament Test^®^ between the two groups. The vibrational sensing on the hallux was intact; however, on the radius on both sides, the vibrational sensing was significantly impaired in the T1DM group compared to the controls with 128 Hz Rydel-Seiffer graduated tuning fork test (**Figure 2**). The Tiptherm^®^-test also identified significant temperature sensitivity impairment in T1DM patients (11 sensing failures *vs.* 1, p < 0.001). In addition, the neuropathic complaints were significantly more frequently present in the T1DM patient group than in the controls (9 *vs.* 0, p < 0.01).

**Table 5:** Peripheral sensory function testing by Neurometer^®^ assessing the threshold of the current sensations at the median and peroneal nerves at three different stimulating frequencies (2000, 250, and 5 Hz).

	T1DM patients (n = 29)	Controls (n = 30)	p value
**NM2000**	188 ± 93	166 ± 86	0.353
**NM250**	85 ± 78	56 ± 38	0.078
**NM5**	50 ± 53	34 ± 28	0.154
**NP2000**	266 ± 122	270 ± 102	0.898
**NP250**	158 ± 104	121 ± 67	0.105
**NP5**	95 ± 76	84 ± 45	0.525

The data are presented as mean ± SD. NM2000, current perception threshold (CPT) value of the median nerve at stimulating frequency of 2,000 Hz; NM250, CPT value of the median nerve at stimulating frequency of 250 Hz; NM5, CPT value of the median nerve at stimulating frequency of 5 Hz; NP2000, CPT value of the peroneal nerve at stimulating frequency of 2,000 Hz; NP250, CPT value of the peroneal nerve at stimulating frequency of 250 Hz; NP5, CPT value of the peroneal nerve at stimulating frequency of 5 Hz.

### Correlations Between Studied Parameters

Diabetes duration (12.2 ± 5.8 years) did not correlate with the results of the cardiovascular reflex tests (HRRDB: r: −0.225, p = 0.242; 30/15 ratio: r: −0.099, p=0.610; VR: r: −0.138, p=0.475, SBPRSU: r: 0.128, p=0.507), the HbA1c level (r: 0.163, p=0.458), or the diastolic blood pressure in T1DM patients (r: 0.309, p=0.103). In addition, no correlation was found between DM duration and the results of the vibration testing by Rydel-Seiffer graduated tuning fork on the right (r: −0.158, p = 0.415) and left (r: −0.162, p = 0.403) radius.

In the whole observed population (diabetic and control subjects together), a significant correlation was found between the HbA1c level and the Valsalva ratio (n = 44, r: −0.483, p = 0.001) and diastolic blood pressure (n = 44, r: 0.352, p = 0.019). Furthermore, a borderline relationship was found between the results of the Tiptherm^®^-tests and the HbA1c level (normal Tiptherm^®^-tests: n = 35, HbA1c: 6.6 ± 1.9% *vs.* abnormal Tiptherm^®^-tests n = 8, HbA1c: 8.9 ± 2.7%, p = 0.051).

## Discussion

In this young T1DM population, at the time of transition from pediatric care to adult-oriented health care system, cardiovascular autonomic neuropathy or cardiac morphological disorders were not found. However, besides intact cardiovascular autonomic and cardiac conditions, peripheral sensory neurological impairments were detected with the 128 Hz Rydel-Seiffer graduated tuning fork test and the Tiptherm^®^-test, along with more severe neuropathic complaints in the T1DM patient group than in the controls.

As poor glycemic control is the most important risk factor for the development of diabetic peripheral neuropathy (DPN) (21, 24, 28, 50, 51), the relatively low rate of DPN among our studied young T1DM patients reflects a good pediatric diabetes care. Early symptoms and signs of peripheral sensory neuropathy have been reported in 23% of children with T1DM by Barkai et al. in 1998 (24); therefore, we expected a higher rate of DPN impairment in our young T1DM group at the time of transition. However, the DCCT (Diabetes Control Complications Trial) Research group found in 1993 that intensive insulin therapy delayed the onset and slowed the progression of DPN by 60% (52), and thus since then the therapeutic management and possibilities (insulin pump therapy, continuous blood glucose monitoring, application of new generation analogue insulins) have been dramatically improved. In our T1DM population, 19 subjects from 29 were on insulin pump therapy at the time of the neuropathic assessment, two of them with CGMS. The therapeutic efficacy in this population was followed by measuring HbA1c level, as Time-In-Range (TIR) monitoring was not accessible until the last few years and it is also well-known that the patients in adolescence are poorly compliant with a sensor therapy. After the transition of the T1DM patients, in the adult-oriented diabetes care in the terms of multiple injections or pump therapy, none of the patients’ therapy has changed over the past year; however, since then nine more patients started to use CGMS.

According to ISPAD (International Society for Pediatric and Adolescent Diabetes) Clinical Practice Consensus Guidelines released in 2018, the screening for peripheral neuropathy should start from age 11 years with 2 to 5 years of diabetes duration and it should be performed annually thereafter (53). Assessment of sensation, vibration, reflexes in the feet for peripheral neuropathy, and orthostatic tests, heart rate variability for cardiac autonomic neuropathy have all been suggested (53). Our study presented a significantly higher prevalence of DPN among T1DM subjects compared to healthy volunteers, and these results confirm a need for early screening and better risk factor management as soon as possible. Interestingly, we found reduced vibration sensation in young T1DM patients in the upper limb and not in the lower limb, which is a rather unusual finding (**Figure 2**). The finding of reduced vibration sensation in young T1DM patients *per se* is not surprising as the large-fiber dysfunction appears first in diabetic length-dependent peripheral neuropathy. However, peripheral neuropathies may affect different types of nerve fibers to different degrees, and the usual concept is that sensory nerves in the lower limb are predominantly affected and symptoms begin at the terminal of the longest nerves in diabetes mellitus (54). On the other hand, the proper diagnosis of diabetic peripheral sensory neuropathy, mostly at the early stages, remains challenging, and several recent reviews summarize the current approaches and suggest strategies to further improve diagnostic testing (55, 56).

The majority of studies on diabetes consider peripheral sensorimotor neuropathy of the lower extremities, and only a few, mostly sensory and motor nerve conduction velocity studies have examined sensory function in the hands (57–61).

The examination of the vibration perception can be useful to detect diabetic peripheral sensory neuropathy at an early stage, and it can be easily assessed by a 128 Hz tuning fork (54). Normative values of vibration perception thresholds in finger pulps and metatarsal heads have been published in healthy children and adolescents (62) and in adults (63). A recent study by Abraham et al. (64) found impaired vibration perception both in the fingers and in the toes among patients with type 1 and type 2 diabetes mellitus; however, the difference was significant only in the toes compared to healthy controls. The study by Ising et al. (65) investigated vibration sensation among children and adolescents with type 1 diabetes, and they also found that impaired vibrotactile sense was more common in the foot than in the hand. However, in few cases of this study, concurrent impaired vibrotactile sense was found in the hand and one subject presented with impaired vibrotactile sense in the hand without having impaired sense in the foot at the same time.

Due to the relative paucity of data on this issue, and given the unusual nature of our present findings on reduced vibration sensation in the upper but not in the lower limbs in young T1DM patients, further studies are warranted to confirm and/or extend these results.

According to recent guidelines (13, 66), it is recommended to assess cardiovascular autonomic function upon diagnosis in type 2 diabetes and within 5 years of diagnosis in type 1 diabetes followed by yearly repeated tests. Established risk factors for CAN are mainly the lack of proper glycemic control in T1DM and, in addition, dyslipidemia, hypertension, obesity in T2DM. Furthermore, glycemic variability (67), oxidative stress, aging-related neuronal dysfunction and death, inflammation, and genetic biomarkers also play important roles in the pathogenesis of CAN (10, 13). Therefore, the intensive glycemic control as early as possible in T1DM is essential to prevent or delay the development of CAN, and beside glycemic control, multifactorial interventions (e.g., lifestyle modification, pharmacological therapy) might be effective in T2DM (13, 14, 68). The EURODIAB IDDM Study and the EURODIAB Prospective Study clearly prove that apart from glycemic control, the classic cardiovascular risk factors (high triglyceride level, body mass index, smoking, and hypertension) are all risk factors of autonomic neuropathy as well (26, 69).

Two risk factors are common in both T1DM and T2DM: the lack of proper glycemic control and the duration of diabetes; however, the most characteristic difference regarding CAN in T1DM and T2DM is the fact that the signs and symptoms appear later in T1DM than in T2DM. This is likely due to the longer duration of the metabolic abnormalities (dysglycemia) and the cascade of multiple complex mechanisms and pathogenic pathways that induce oxidative stress and autonomic neuronal dysfunction in diabetes, which occur already prior to the diagnosis of T2DM (70–72). The pathogenesis of type 2 diabetes is more complex than in type 1 diabetes, and the abovementioned cardiovascular risk factors are much more frequently present, which explains the higher prevalence of autonomic neuropathy in type 2 diabetic patients. With a mean diabetes duration of 12.2 ± 5.8 years in our study group, CAN would have been definitely expected. The reasons for the lack of the impairment might be multifactorial, including the young age, the efficient pediatric diabetes care, and the ideal body weight of the studied diabetic population achieved by sport.

Among the young T1DM patients, we found significantly higher comorbidity with hypertension (in six diabetic patients) with a significantly increased diastolic blood pressure without detectable cardiac morphologic differences or cardiovascular autonomic neuropathy compared to controls, and in the whole observed population (diabetic and control subjects together), a significant correlation was found between HbA1c level and diastolic blood pressure. This finding is consistent with previous studies on diastolic blood pressure in childhood diabetes (73–75).

It is well-known that hypomagnesemia is common in poorly controlled and chronically treated diabetic patients (76, 77). Several previous studies—consistent with our present finding—demonstrated lower serum magnesium level in young T1DM patients (78–84). A recent systematic review and meta-analysis by Rodrigues et al. (85) showed an association between reduced levels of magnesium and poor glycemic control in patients with T1DM, potentially contributing to the early development of cardiovascular complications.

In summary, at the time of transition from pediatric to adult-oriented health care in this young T1DM population, cardiovascular autonomic neuropathy and cardiac morphological differences could not be found. However, peripheral sensory neurological impairments with several modalities were detected among young type 1 diabetic patients. The results may provide important feedback to pediatric diabetes care and a remarkable baseline reference point for further follow-up in adult diabetes care.

## Limitations

Continuous involvement of young T1DM patients following their entrance to the adult healthcare system and further enlargement of this specific study population is planned in the future. In addition, an annual follow-up of the already involved subjects is designed to estimate the progress of their neuropathic state. The listed medicines might also have an impact on the test results. Due to the low number of T1DM patients on oral antihypertensive therapy, a statistical analysis has not been feasible to perform yet. The echocardiographic examinations and the collection of the blood and urine samples due to logistical reasons were not possible from all the patients and controls.

## Data Availability Statement

The raw data supporting the conclusions of this article will be made available by the authors, without undue reservation.

## Ethics Statement

The studies involving human participants were reviewed and approved by Hungarian Medical Research Council. The patients/participants provided their written informed consent to participate in this study.

## Author Contributions

AV, ÁM, ÉC, KS, IB, PK, AO, TV, and CL had substantial contributions to the conception of the work and design of the paper and read and approved the final manuscript. CP, DU-P, AN, MS, AO, and AV contributed to the measurements and analyses of data. AV, IB, PK, TV, AO, and CL drafted the paper or revised it critically for important intellectual content. All authors contributed to the article and approved the submitted version.

## Funding

This work was supported by the Ministry of Human Capacities of Hungary (EFOP-3.6.3-VEKOP-16-2017-00009), the Hungarian Diabetes Association, the University of Szeged Open Access Fund (Grant No. 5455), and by the National Research, Development and Innovation Office (NKFIH-K-128851).

## Conflict of Interest

The authors declare that the research was conducted in the absence of any commercial or financial relationships that could be construed as a potential conflict of interest.

## Publisher’s Note

All claims expressed in this article are solely those of the authors and do not necessarily represent those of their affiliated organizations, or those of the publisher, the editors and the reviewers. Any product that may be evaluated in this article, or claim that may be made by its manufacturer, is not guaranteed or endorsed by the publisher.
